# Patterns of mortality risk among patients with substance use disorder: an opportunity for proactive patient safety?

**DOI:** 10.1186/s12888-022-04437-6

**Published:** 2022-12-07

**Authors:** Jakob Svensson, Johan Bergström, Martin Kåberg, Per Becker

**Affiliations:** 1 Division of Risk Management and Societal Safety, Lund University, Box 118, SE-22100 Lund, Sweden; 2 Department of Global Public Health, Karolinska Institutet, Stockholm, Sweden; 3 Unit for Environmental Sciences and Management, North-West University, Private Bag X6001, 2520 Potchefstroom, South Africa

**Keywords:** Patient safety, Psychiatry, Emergency ward, Substance use disorder, Mortality, Risk

## Abstract

**Background:**

Patients with substance use disorder (SUD) suffer from excess mortality compared to the overall population. This study aims to identify patterns in death rates among patients with SUD visiting a SUD emergency ward and to explore whether this knowledge can be used as input to identify patients at risk and increase patient safety.

**Methods:**

Hospital visit data to a SUD emergency ward were collected between 2010 and 2020 through medical records. Data included gender, age, SUD diagnosis, and the time of death. The Kruskal-Wallis rank sum test was used to test between ordinal variables, and risk ratio was used to quantify the difference in mortality risk. All statistical tests were two-sided, with a 95% confidence interval and a minimum significance level of 0.05.

**Results:**

The male patients in the study group had 1.41–1.59 higher mortality risk than the female patients. The study revealed an average death rate of 0.14 among all patients during the study period. Although patients with a diagnosed alcohol use disorder constituted 73.7% of the cohort, having an opioid use disorder or sedative hypnotics use disorder was associated with the highest death rates; 1.29–1.52 and 1.47–1.74 higher mortality risk than those without such diagnoses.

**Conclusion:**

This study demonstrates that data from visits to SUD emergency wards can be used to identify mortality risk factors, such as gender, type of diagnosis, number of diagnoses, and number of visits to the SUD emergency ward. Knowledge about patterns of patient visits and mortality risk could be used to increase patient safety through a decision support tool integrated with the electronic medical records. An improved system for early detection of increased mortality risk offers an opportunity for an adaptive patient safety system.

## Background

Patients with substance use disorder (SUD) suffer from excess mortality compared to the overall population [1–3], and SUD often requires long-term strategic treatment addressing contextual risk factors, life skills training, and social support [4–7]. Studying patterns in patients revisiting healthcare provides an opportunity to detect risk factors for premature mortality in specific patient groups [8]. In a follow-up study over three decades of a cohort of substance users in Sweden, predictors of increased risk of drug-related death were associated with male gender, the use of opiates or barbiturates, and depression and anxiety disorders at first admission [9]. In addition to these risk factors, physical conditions such as chronic lung conditions and hepatitis C have been associated with readmission risk in patients with SUD [10]. This knowledge provides a foundation to increase patient safety for SUD emergency ward patients. This study is positioned within contemporary patient safety science, seeing safety as an emergent and path-dependent property of everyday variability in complex healthcare environments [11–13]. This idea suggests that normal organizational processes contain information about organizational failure and success. This study aims to identify patterns in the death rate among patients with SUD visiting a SUD emergency ward and to explore whether this knowledge can be used as input to identify patients at risk and increase patient safety.

## Methods

### Data collection

Data were collected from visits to a SUD emergency ward in Stockholm County, Sweden. The emergency ward delivers a transient patient care model, which means that patients are triaged to the required level of care (observation, inpatient-care, somatic or psychiatric care, or discharged home with or without an outpatient follow-up) based on vital parameters, active psychosis, or suicidal intention (yes or no). Healthcare personnel estimates a risk value for the patient’s substance use and somatic and psychiatric health, and the patient is offered treatment and care based on this risk assessment. Anonymized data were extracted from medical records, including information about gender, age, and SUD diagnosis. Hospital visit data to the SUD emergency ward were collected between 2010 and 2020. Data also included mortality and time of death. Clinical doctors classified all SUD diagnoses following the standardized protocol of the studied clinic.

**Table 1:** List of included mental and behavioral disorders due to psychoactive substance use

ICD-code	SUD
F10	Alcohol
F11	Opioids
F12	Cannabinoids
F13	Sedative hypnotics
F14	Cocaine
F15	Other stimulants, including caffeine
F16	Hallucinogens
F17	Tobacco
F18	Volatile solvents
F19	Multiple drug use and use of other psychoactive substances

Inclusion criteria were a visit to the SUD emergency ward and a SUD diagnosis. The psychiatric taxonomy of ICD-10 was used to differentiate between types of SUD, and diagnoses included F10-F19 (Table 1). Information on the time of death was included nine months after the study period, to October 2021. All participants were registered through their unique Swedish personal identity numbers. Those without a personal identity number were excluded since reoccurring visits and mortality could not be evaluated within this group. Consequently, 5.9% of the total number of visits to the emergency ward were excluded.

### Statistical analysis

**Fig. 1:**
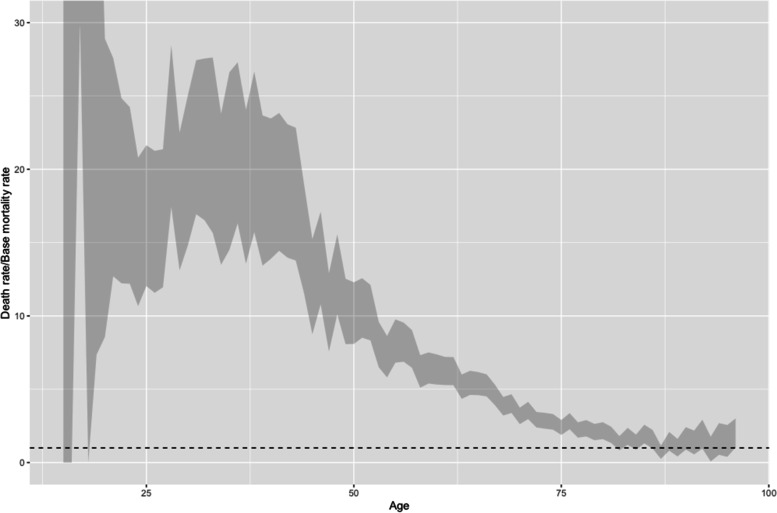
95% confidence interval of the ratio between patient death rate and base mortality for Stockholm County per year of age. Results over 1 represent excess mortality (dotted line)

The death rates for each year of age among the patients were analyzed in relation to the base mortality rate for Stockholm County during the same period (Fig. 1). Non-overlapping confidence intervals (95%) for the death rates of different groups divided over binary categorical variables were interpreted as indicating a statistically significant difference in death rates between the groups. For ordinal variables, the Kruskal-Wallis rank sum test was used as a nonparametric test to check if there were any ordinal differences in death rate. Risk ratio was used to quantify the differences in mortality risk across binary categorical variables or between each step in ordinal variables. Patients could have received multiple diagnoses during a visit and different diagnoses during different visits. The analysis focused on a particular diagnosis (Table 3) or combination of diagnoses (Table 5), ignoring any other potential diagnoses, and sometimes focusing on the differences in death rates between having only one particular diagnosis and combining it with different numbers of other diagnoses (Table 4). Associations between death rate and all diagnoses of SUD were statistically tested. All statistical tests were two-sided, with a 95% confidence interval and a minimum significance level of 0.05.

## Results

### Patient characteristics

Table 2 summarizes the main characteristics of the patients in the study group. The study reveals an average death rate of 0.14 among all patients during the study period (Table 2).

**Table 2:** Characteristics of patients included in the study

Total number of visits 2010–2020	157,200
Proportion of female and male	31.22% and 68.78%
Year of birth (mean)	1921–2004 (1973)
Patients with F10 diagnosis (%)	27,959 (73.66%)
Patients with F11 diagnosis (%)	2903 (7.65%)
Patients with F12 diagnosis (%)	1957 (5.16%)
Patients with F13 diagnosis (%)	2031 (5.35%)
Patients with F14 diagnosis (%)	550 (1.45%)
Patients with F15 diagnosis (%)	2582 (6.80%)
Patients with F16 diagnosis (%)	89 (0.235%)
Patients with F17 diagnosis (%)	3 (0.0079%)
Patients with F18 diagnosis (%)	15 (0.0395%)
Patients with F19 diagnosis (%)	8964 (23.62%)
Number of types of diagnoses (mean)	1–7 (1.24)
Total number of visits	157,200 visits
Visits per patient (mean)	1-449 (4.14)
Overall death rate during study period	0.1404
Total number of patients 2010–2020	37,959 patients

### Gender, age, and type of diagnosis

Males had a 1.41–1.59 higher mortality risk than females during the study period (Table 3). While the death rate increased with age, the excess mortality varied substantially over age (Fig. 1). For instance, 25-year-old patients had 12–22 times excess mortality, and 50-year-old patients had 8–12,5 excess mortality during the study period, while not being statistically significant among those in their early 80s’. The confidence interval overlapping the base mortality rate (dotted line) was interpreted as the difference not being statistically significant. The sample was too small for the youngest patients to generate informative results.

The death rates for single diagnoses in the ICD-10 system (F10-F19), and multiple diagnoses, are presented in Table 3. The death rates for a specific diagnosis are presented over the death rates for the patients not having that particular diagnosis. Non-overlapping confidence intervals of the death rates signify statistically significant differences between the pairs. Table 3 also includes risk ratios to quantify the difference for each step between categories and provide the level of significance. It is worth noting that patients with a sedative hypnotics diagnosis have the highest death rate (0.20–0.24) in the study period, which corresponds to a 1.47–1.74 higher mortality risk than patients not having that diagnosis.

**Table 3:** Associations between death rate and gender, types of diagnosis, and number of types of diagnosis

Variables	Death rate (95% CI)	Risk ratio (95% CI)
Gender	Men: 0.1567 (0.1522–0.1611) Women: 0.1046 (0.0991–0.1101)	1.497 (1.411–1.590) *p* < 0.0001
F10 diagnosis (alcohol)	F10: 0.1504 (0.1462–0.1546) Not F10: 0.1126 (0.1064–0.1188)	1.335 (1.256–1.420) *p* < 0.0001
F11 diagnosis (opioids)	F11: 0.1908 (0.1765–0.2051) Not F11: 0.1362 (0.1326–0.1398)	1.401 (1.294–1.517) *p* < 0.0001
F12 diagnosis (cannabinoids)	F12: 0.0761 (0.0644–0.0879) Not F12: 0.1439 (0.1403–0.1475)	0.529 (0.452–0.619) *p* < 0.0001
F13 diagnosis (sedative hypnotics)	F13: 0.2176 (0.1997–0.2356) Not F13: 0.1360 (0.1325–0.1396)	1.600 (1.467–1.744) *p* < 0.0001
F14 diagnosis (cocaine)	F14: 0.0655 (0.0447–0.0862) Not F14: 0.1415 (0.1380–0.1450)	0.463 (0.337–0.635) *p* < 0.0001
F15 diagnosis (other stimulants, incl caffeine)	F15: 0.1150 (0.1027–0.1273) Not F15: 0.1423 (0.1386–0.1459)	0.809 (0.724–0.902) *p* = 0.0001
F16 diagnosis (hallucinogens)	F16: 0.0337 (-0.0045-0.0719) Not F16: 0.1407 (0.1372–0.1442)	0.240 (0.079–0.729) *p* = 0.0037
*F17 diagnosis (tobacco)*	*F17: 0 (0–0)* *Not F17: 0.1404 (0.1369–0.1439)*	*0 (0)* *p = 0.4839*
*F18 diagnosis (volatile solvents)*	*F18: 0.2667 (0.0132–0.5202)* *Not F18: 0.1404 (0.1369–0.1439)*	*1.900 (0.821–4.399)* *p = 0.1592*
F19 diagnosis (multiple drug use of other psychoactive substances)	F19: 0.1399 (0.1327–0.1471) Not F19: 0.1406 (0.1366–0.1446)	*0.995 (0.938–1.055)* *p = 0.8708*
Number of types of diagnoses	1: 0.1348 (0.1310–0.1385) 2: 0.1562 (0.1450–0.1673) 3: 0.1904 (0.1712–0.2096) 4 or more: 0.2021 (0.1688–0.2355) p < 0.0001^i^	Increase from 1 to 2: 1.159 (1.073–1.251) *p* = 0.0002 Increase from 2 to 3: 1.219 (1.078–1.380) *p* = 0.0018 *Increase from 3 to 4: 1.062 (0.875–1.288)* *p = 0.5454*

### Combination of diagnostic categories

The analyses of diagnostic categories show varying increases in mortality risk when adding additional diagnoses to the different types of diagnoses (Table 4). The study confirms the high risk for mortality for male patients with SUD and that alcohol use in combination with opioids or sedative hypnotics increases the death rate (Table 5). While all types of diagnoses were associated with increased death rates, the more additional SUD diagnoses the patients had, the stepwise analyses of risk ratio struggle to establish statistically significant quantifications of how much mortality risk increased each step. This issue is partly explained by the inherent process of subdividing the sample for each step. Since the sample was too small for patients with a hallucinogen (F16), tobacco (F17), or volatile solvents (F18) diagnosis to provide any informative results, they are removed from Tables 4 and 5.

**Table 4:** Associations between death rate for each type of diagnosis and number of additional types of diagnoses

**Variables**	**Death rate (95% CI)**	**Risk ratio (95% CI)**
**Only F10 (alcohol) or with other types of diagnoses**	Only F10: 0.1472 (0.1427-0.1516) F10+1: 0.1626 (0.1469-0.1783) F10+2: 0.1853 (0.1608-0.2098) F10+3 or more: 0.1939 (0.1560-0.2317) *p* < 0.0001^i^	*+1/only F10: 1.105 (0.999-1.222)* *p = 0.0548* *+2/+1: 1.140 (0.968-1.342)* *p = 0.119* *+3 or more/+2: 1.046 (0.827-1.323)* *p = 0.707*
**Only F11 (opioids) or with other types of diagnoses**	Only F11: 0.1194 (0.0985-0.1404) +1 type: 0.2092 (0.1828-0.2355) +2 types: 0.2349 (0.2033-0.2665) +3 or more: 0.2405 (0.1968-0.2843) *p* < 0.0001^i^	+1/only F11: 1.751 (1.411-2.173) *p* < 0.0001 *+2/+1: 0.123 (0.934-1.350)* *p = 0.217* *+3 or more/+2: 1.024 (0.817-1.283)* *p = 0.836*
**Only F12 (cannabinoids) or with other types of diagnoses**	Only F12: 0.0433 (0.0305-0.0561) +1 type: 0.0713 (0.0488-0.0938) +2 types: 0.1307 (0.0912-0.1703) +3 or more: 0.1709 (0.1181-0.2236) *p* < 0.0001^i^	+1/only F12: 1.646 (1.069-2.536) *p* = 0.0227 +2/+1: 1.834 (1.187-2.834) *p* = 0.0058 *+3 or more/+2: 1.307 (0.851-2.007)* *p = 0.2217*
**Only F13 (sedative hypnotics) or with other types of diagnoses**	Only F13: 0.1454 (0.1145-0.1764) +1 type: 0.2450 (0.2090-0.2810) +2 types: 0.2413 (0.2071-0.2755) +3 or more: 0.2359 (0.1926-0.2792) *p* < 0.0001^i^	+1/only F13: 1.685 (1.302-2.180) *p* < 0.0001 *+2/+1: 0.985 (0.804-1.207)* *p = 0.884* *+3 or more/+2: 0.978 (0.776-1.232)* *p = 0.8477*
**Only F14 (cocaine) or with other types of diagnoses**	Only F14: 0.0303 (0.0080-0.0526) +1 type: 0.0629 (0.0227-0.1032) +2 types: 0.0755 (0.0244-0.1266) +3 or more: 0.1714 (0.0809-0.2619) *p* = 0.0005^i^	*+1/only F14: 2.077 (0.791-5.454)* *p = 0.1301* *+2/+1: 1.199 (0.479-3.005)* *p = 0.6988* +3 or more/+2: 2.271 (0.979-5.273) *p* = 0.0503
**Only F15 (other stimulants, incl caffeine) or with other types of diagnoses**	Only F15: 0.0829 (0.0642-0.1017) +1 type: 0.1055 (0.0839-0.1272) +2 types: 0.1433 (0.1157-0.1710) +3 or more: 0.1619 (0.1233-0.2006) *p* < 0.0001^i^	*+1/only F15: 1.273 (0.938-1.726)* *p = 0.1204* +2/+1: 1.358 (1.025-1.798) *p* = 0.0322 *+3 or more/+2: 1.130 (0.832-1.534)* *p = 0.4349*
**Only F19 (multiple drug use of other psychoactive substances) or with other types of diagnoses**	Only F19: 0.0992 (0.0897-0.1088) +1 type: 0.1513 (0.1387-0.1638) +2 types: 0.1928 (0.1730-0.2126) +3 or more: 0.2051 (0.1713-0.2389) *p* < 0.0001^i^	+1/only F19: 1.524 (1.342-1.731) *p* < 0.0001 +2/+1: 1.274 (1.117-1.454) *p* = 0.0003 *+3 or more/+2: 1.064 (0.876-1.291)* *p = 0.5334*

i = Kruskal-Wallis rank sum test

The difficulty of establishing statistical significance is not present when analyzing patterns of mortality risk for combinations of types of diagnoses (Table 5). These results identify the combinations with the highest mortality and quantify the increase in mortality risk when adding a particular type of diagnosis. It is worth noting the staggering death rate of patients with at least both opioids and sedative hypnotics diagnoses (0.24–0.30), as well as the 1.42–1.93 increase in mortality risk between patients having an opioids diagnosis and adding a sedative hypnotics diagnosis while having a sedative hypnotics diagnosis and adding an opioids diagnosis increases mortality risk by 1.21–1.68.

**Table 5:** Associations between death rate and combinations of types of diagnoses

Variables	Death rate (95% CI)	Risk ratio (95% CI)
**F10 diagnosis and F11 diagnosis or not**	F10 + F11: 0.2268 (0.1941–0.2594) F10, not F11: 0.1486 (0.1444–0.1528)	1.526 (1.318–1.767) *p* < 0.0001
**F10 diagnosis and F13 diagnosis or not**	F10 + F13: 0.2521 (0.2204–0.2836) F10, not F13: 0.1476 (0.1434–0.1519)	1.707 (1.502–1.941) *p* < 0.0001
**F10 diagnosis and F19 diagnosis or not**	F10 + F19: 0.1728 (0.1586–0.1870) F10, not F19: 0.1479 (0.1435–0.1523)	1.168 (1.071–1.275) *p* = 0.0005
**F11 diagnosis and F10 diagnosis or not**	F11 + F10: 0.2268 (0.1941–0.2594) F11, not F10: 0.1808 (0.1649–0.1966)	1.254 (1.060–1.484) *p* = 0.0091
**F11 diagnosis and F13 diagnosis or not**	F11 + F13: 0.2713 (0.2378–0.3047) F11, not F13: 0.1661 (0.1506–0.1816)	1.653 (1.416–1.926) *p *< 0.0001
**F11 diagnosis and F19 diagnosis or not**	F11 + F19: 0.2245 (0.2046–0.2443) F11, not F19: 0.1438 (0.1240–0.1636)	1.561 (1.325–1.838) *p* < 0.0001
**F12 diagnosis and F10 diagnosis or not**	F12 + F10: 0.1267 (0.0927–0.1607) F12, not F10: 0.0643 (0.0522–0.0764)	1.970 (1.421–2.730) *p* < 0.0001
**F12 diagnosis and F11 diagnosis or not**	F12 + F11: 0.2039 (0.1392–0.2687) F12, not F11: 0.0654 (0.0540–0.0768)	3.120 (2.178–4.468) *p* < 0.0001
**F12 diagnosis and F13 diagnosis or not**	F12 + F13: 0.1880 (0.1207–0.2552) F12, not F13: 0.0680 (0.0564–0.0795)	2.765 (1.868–4.092) *p* < 0.0001
**F12 diagnosis and F15 diagnosis or not**	F12 + F15: 0.1123 (0.0748–0.1498) F12, not F15: 0.0702 (0.0580–0.0824)	1.600 (1.1002–2.327) *p* = 0.0145
**F12 diagnosis and F19 diagnosis or not**	F12 + F19: 0.1196 (0.0963–0.1430) F12, not F19: 0.0495 (0.0372–0.0617)	2.418 (1.766–3.312) *p* < 0.0001
**F13 diagnosis and F10 diagnosis or not**	F13 + F10: 0.2521 (0.2205–0.2836) F13, not F10: 0.1983 (0.1766-0.2200)	1.271 (1.077–1.501) *p* = 0.0049
**F13 diagnosis and F11 diagnosis or not**	F13 + F11: 0.2713 (0.2378–0.3047) F13, not F11: 0.1905 (0.1695–0.2115)	1.424 (1.207–1.679) *p *< 0.0001
**F13 diagnosis and F19 diagnosis or not**	F13 + F19: 0.2348 (0.2099–0.2597) F13, not F19: 0.1967 (0.1709–0.2225)	1.193 (1.008–1.412) *p* = 0.0387
**F14 diagnosis and F10 diagnosis or not**	F14 + F10: 0.1039 (0.0552–0.1526) F14, not F10: 0.0505 (0.0288–0.0721)	2.057 (1.095–3.864) *p* = 0.0231
**F14 diagnosis and F13 diagnosis or not**	F14 + F13: 0.2000 (0.0606–0.3394) F14, not F13: 0.0563 (0.0363–0.0763)	3.552 (1.676–7.527) *p* = 0.0009
**F14 diagnosis and F15 diagnosis or not**	F14 + F15: 0.1346 (0.0679–0.2013) F14, not F15: 0.0493 (0.0292–0.0695)	2.729 (1.446–5.151) *p* = 0.0016
**F14 diagnosis and F19 diagnosis or not**	F14 + F19: 0.1029 (0.0609–0.1450) F14, not F19: 0.0434 (0.0218–0.0649)	2.375 (1.253–4.501) *p* = 0.0064
**F15 diagnosis and F11 diagnosis or not**	F15 + F11: 0.1922 (0.1479–0.2365) F15, not F11: 0.1046 (0.0920–0.1172)	1.837 (1.418–2.380) *p *< 0.0001
**F15 diagnosis and F13 diagnosis or not**	F15 + F13: 0.1754 (0.1296–0.2212) F15, not F13: 0.1080 (0.0954–0.1207)	1.623 (1.221–2.158) *p *< 0.0011
**F15 diagnosis and F19 diagnosis or not**	F15 + F19: 0.1381 (0.1202–0.1561) F15, not F19: 0.0865 (0.0703–0.1027)	1.597 (1.272–2.006) *p* < 0.0001
**F19 diagnosis and F10 diagnosis or not**	F19 + F10: 0.1728 (0.1586–0.1870) F19, not F10: 0.1254 (0.1172–0.1336)	1.378 (1.241–1.530) *p* < 0.0001
**F19 diagnosis and F11 diagnosis or not**	F19 + F11: 0.2245 (0.2046–0.2443) F19, not F11: 0.1202 (0.1127–0.1277)	1.867 (1.676–2.081) *p* < 0.0001
**F19 diagnosis and F13 diagnosis or not**	F19 + F13: 0.2348 (0.2099–0.2597) F19, not F13: 0.1264 (0.1190–0.1338)	1.857 (1.646–2.096) *p* < 0.0001

Table 6 presents the effects on the death rate of the combinations with the highest mortality of two types of diagnoses when adding additional types of diagnoses. There was no statistically significant effect for any combinations (Table 6).

**Table 6:** Associations between death rate and particular combinations of types of diagnoses, with additional types of diagnoses or not

Variables	Death rate (95% CI)
**Only F10 and F11 or with other types of diagnoses**	Only F10 + F11: 0.1917 (0.1202–0.2631) + 1 type: 0.2290 (0.1778–0.2802) + 2 or more: 0.2411 (0.1880–0.2942) *p* = 0.564^i^
**Only F10 and F13 or with other types of diagnoses**	Only F10 + F13: 0.2780 (0.2188–0.3373) + 1 type: 0.2387 (0.1847–0.2927) + 2 or more: 0.2424 (0.1904–0.2945) *p* = 0.5608^i^
**Only F11 and F13 or with other types of diagnoses**	Only F11 + F13: 0.3261 (0.2285–0.4237) + 1 type: 0.2745 (0.2242–0.3245) + 2 or more: 0.2500 (0.1993–0.3007) *p* = 0.3567^i^
i = Kruskal-Wallis rank sum test	

### Death rate and number of emergency ward visits

**Fig. 2:**
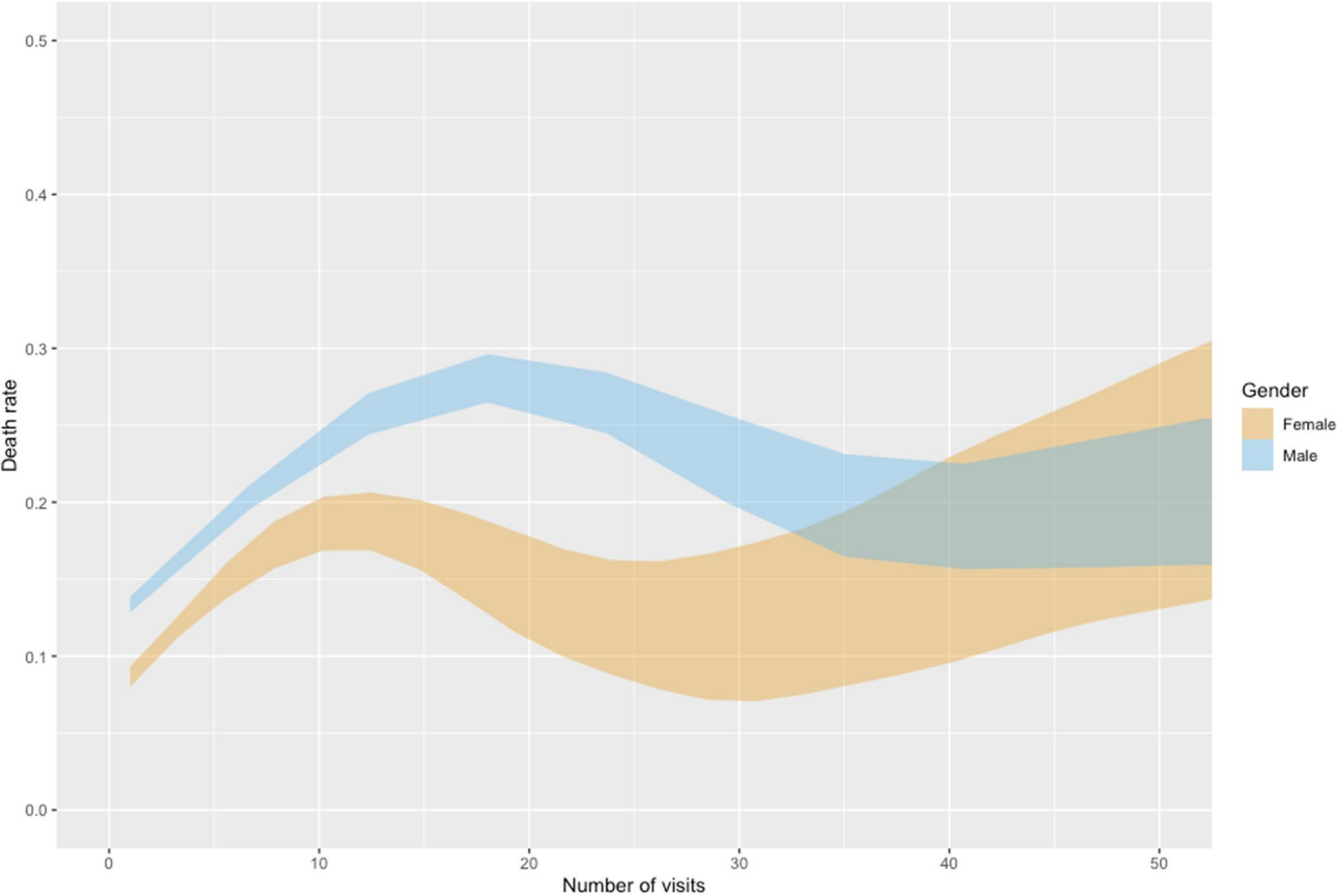
Death rate and number of visits for men and women, regardless of diagnoses

**Fig. 3:**
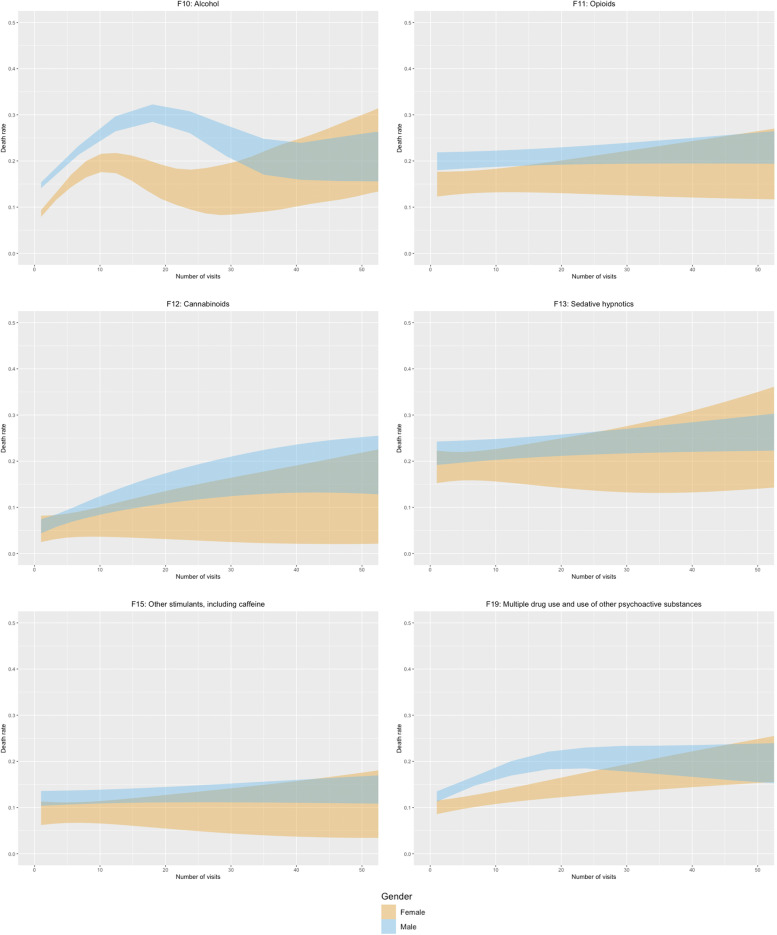
Death rate and number of visits for men and women per type of diagnosis

The findings in this study demonstrate that the number of different SUD diagnoses increases mortality risk and that the number of visits to the SUD emergency ward indicates a higher risk for mortality. Figures 2 and 3 present the 95% confidence intervals for death rate, and non-overlapping confidence intervals denote statistically significant differences. Figure 2 reveals patterns in the associations between death rate, number of visits, and gender, with a significant increase in death rate up to around 12 visits for women and 18 visits for men, before decreasing again. No difference between men and women was noticed after 32 visits, and the widening confidence intervals hamper any further conclusions concerning any trends in death rate (Fig. 2). However, these patterns are almost exclusively driven by patients with an alcohol diagnosis (Fig. 3), who comprise nearly three-quarters of the study population (Table 1). Men with an alcohol diagnosis reach an even higher average death rate around 18 visits, than the combination of opioids and sedative hypnotics diagnoses.

The result showed stability in mortality risk over time for patients with only an opioids or sedative hypnotics diagnosis. None of the other types of diagnoses exhibited the same pattern in death rate as alcohol (Fig. 3). In addition to alcohol, only opioids and polysubstance use diagnosis (F19 diagnosis) demonstrated statistically significant differences between women and men within specific numbers of visits, and only patients with polysubstance use diagnosis and male patients with a cannabinoids diagnosis showed an unequivocal increase in death rate for any span of visits (Fig. 3). Results for Cocaine (F14), Hallucinogens (F16), Tobacco, (F17)and Volatile solvents (F18) were inconclusive due to small sample sizes.

## Discussion

### Patterns of mortality risk

#### Basic demographic patterns

The study showed between 1.41 and 1.59 higher mortality risk among male patients with SUD than female patients with SUD within the study period (Table 3). This pattern suggests gender as a substantial risk factor, which concurs with established theory that the male gender is associated with drug-related premature death [9]. However, such a gendered pattern of risk was only visible for specific diagnoses and certain numbers of visits to the emergency ward, which is further discussed below. It is also interesting to note that while the death rate increases with age, the excess mortality was highest for patients in their late 20s to their early 40s (Fig. 1). Age is, as such, not as straightforward a risk factor as it may initially appear [14].

#### Type of diagnosis

The overall death rate of 0.14 among patients in this study was high but in line with a meta-review of all-cause and suicide mortality among major mental disorders, attributing the highest mortality to SUD diagnoses [15]. Alcohol and illicit drugs generally contribute to premature deaths [1], but our results showed pronounced variation between the different diagnoses. Particularly high death rate was primarily associated with alcohol (0.146–0.155), opioids (0.18–0.21), or sedative hypnotics diagnoses (0.20–0.24), while having a polysubstance use diagnosis was associated with an average death rate (0.13–0.15) and the other diagnoses with lower-than-average death rates in the cohort (Table 3). It is important to note that patients with an alcohol diagnosis constitute 73.66% of the cohort. Alcohol-related death is complex due to its association with violence, suicide, and accidents [1]. The results show that having an opioid and a sedative hypnotics diagnosis in combination was associated with the highest death rates (Table 5). However, substantially lower death rates were found among the patients with only opioids or sedative hypnotics diagnoses (Table 4). This pattern is particularly notable for patients with only an opioids diagnosis, with a death rate below average (0.10–0.14) even when opioids are generally associated with a high mortality on their own [16, 17]. This pattern could be explained by the impact of opioid agonist treatment (OAT), which reduces opioid-related deaths [18, 19]. However, the results indicate that one type of diagnosis has limited explanatory power of the patterns of mortality risk on its own while having multiple and combinations of types of diagnosis require more effective interventions.

### Multiple and combinations of SUD diagnoses

The analyses of diagnostic categories show varying increases in mortality risk when adding additional diagnoses to the different types of diagnostic categories (Table 4). Death rate also increased with the number of different types of diagnoses of the patients (Table 3). For instance, patients with two types of diagnoses had 1.07–1.25 higher mortality risk than patients with only one diagnosis, and patients with three diagnoses had 1.08–1.38 higher mortality risk—regardless of what types of diagnoses they had. Likewise, patients with cannabinoids diagnosis increased mortality risk by 1.07–2.54 with their first additional SUD diagnosis and another 1.19–2.83 with their second diagnosis (Table 4). However, that could not explain why death rates increased with additional types of diagnoses for patients already with the types of diagnosis associated with the highest death rates, such as opioids and sedative hypnotics, by their first additional type of diagnosis (Table 4).

The higher mortality could partly be explained by the increased mortality risk of polysubstance use [1, 9], provided that they use the substances simultaneously and do not get the different types of diagnoses one by one over time. The mortality risk of patients with a polysubstance use diagnosis (F19) is interesting since this diagnosis indicates multiple drug use (Table 4). However, we noted that this diagnosis was associated with a low death rate (0.10–0.11) on its own but with a 1.34–1.73 increase in mortality risk by the first and another 1.12–1.45 increase by the second additional SUD diagnosis. This pattern indicates that the F19 diagnosis may be used as a ‘catch-all’ category for what is not covered by the other types of diagnoses than for polysubstance use.

The explanation of polysubstance use is particularly pertinent to OAT patients with an opioid diagnosis. Although caution must be taken when interpreting these results, patients in OAT programs might still be discharged from methadone treatment due to the use of illicit drugs [20]. Other studies show that discharged OAT patients have a 20 times higher mortality risk than those who remain in the program [18, 19]. This pattern may explain the lower mortality risk among patients with only an opioids diagnosis, even if the study ignores that patients with many types of diagnoses may still be eligible for OAT. However, it also indicates that a zero-tolerance policy, demanding a complete absence of illicit drug use, does not benefit harm-reduction and patient safety.

Our results also revealed the importance of particular combinations of SUD diagnoses for the patterns of mortality risk. The combination of opioids and sedative hypnotics had the highest death rate (0.24–0.30), regardless of what other diagnoses the patients had (Table 5). However, the death rate was equally high among patients with only these two types of diagnoses (0.23–0.42), with no statistically significant difference with additional types of diagnoses. The combination of alcohol and sedative hypnotics was similar, with a similarly increased death rate regardless of the presence of other types of diagnoses or not (Table 5). These results concur with previous studies, showing that use of sedative hypnotics is common among patients with polysubstance use [21] and are also associated with high mortality, including overdose and suicide, with overdose being more likely when used together with opioids and/or alcohol [22]. Our results also indicate a high death rate among patients combining alcohol and opioids (0.20–0.26).

Our results revealed patterns of mortality risk for patients using substances associated with lower death rates in relation to combining them with other types of diagnoses (Table 5). For instance, patients using cannabinoids had an increased mortality risk of 1.42–2.73 if also having an alcohol diagnosis, 2.18–4.47 if also having an opioid diagnosis, and 1.87–4.09 if also having a sedative hypnotics diagnosis. Even more pronounced patterns emerge for users of cocaine (Table 5), with mortality risk increasing from 1.68 up to as much as 7.53 if also having a sedative hypnotics diagnosis. Moreover, it is in combination with the types of diagnoses with the highest mortality that the polysubstance use diagnosis (F19) seems to play a role, e.g. increasing mortality risk 1.33–1.84 for patients with an opioids diagnosis (Table 5).

#### Number of visits

Our results reveal that frequency of visits to the SUD emergency ward was a critical risk factor. While the Swedish action plan for improved healthcare for patients with SUD highlights the emergency ward as crucial for detecting early signals of increased patient risk [23], there may be a need for re-examining what is considered a threshold to trigger such signals. This issue is particularly pertinent for patients with an alcohol diagnosis (Fig. 3), with rapid exacerbation of mortality risk already from the first visit and with men reaching similar death rates as the diagnoses associated with the highest death rate after between four to seven visits (Table 3) and as the deadliest combination of types of diagnoses after between 10 and 13 visits (Table 5) before the alcohol diagnosis have the highest mortality risk of all at around 18 visits. Considering the high proportion of all patients having an alcohol diagnosis, many lives could be saved if the healthcare system could identify multi-visitors at an earlier stage. Patients with a polysubstance use diagnosis (F19) and male patients with a cannabinoids diagnosis would also benefit from being identified earlier. However, mortality is lower in this group compared to the alcohol diagnosis. It is important to note that the death rates for patients with opioids or sedative hypnotics diagnoses seem relatively stable, but on a high level, across the number of visits.

### Patterns of risk as an opportunity for proactive patient safety

The results of this study demonstrate that data from visits to emergency wards for SUD can be used to identify combined mortality risk factors, such as gender, age, type of diagnosis, number of diagnoses, polydrug use, and number of visits to the emergency ward. While previous studies identify several risk factors, such as opioid use on its own, age, or substantial alcohol use [14], the results of this study suggest more complex mechanisms that defy linear thinking and complicate the triage work in the SUD emergency wards. There are also indications of increasing visits placing further pressure on their staff, which has been suggested to negatively impact patient safety [24, 25]. While various strategic interventions are implemented to address internal and external pressures, with the explicit aim of strengthening patient safety, psychiatric emergency wards exist in a complex environment where patient safety measures require continuous adaptation [26, 27]. An improved system for early detection of increased mortality risk offers one opportunity for such adaptation.

This paper contributes to the psychiatric patient safety literature with an increased system-level understanding of how mortality risk emerges over time. The results of this study demonstrate the potential for more systematic use of electronic medical records. Monitoring large datasets has been suggested as an upcoming field for suicide prevention [28], which is relevant in this setting since 14.3% of deaths worldwide can be associated with mental disorders [3]. A decision support tool that is integrated with the electronic medical records could allow clinicians to focus on the present visit of the patient and provide evidence-based input to the triaging of the patient’s required level of care based on automized analysis of how his or her current diagnoses and demographics combine with the medical history in relation to the overall patterns of mortality risk in the county. Thereby incorporating a modern patient safety viewpoint where understanding the system’s current state includes the patients’ journey within the healthcare system over time [13]. Knowledge about patterns of patient visits and mortality risk could thus contribute to a more adaptive patient safety system.

### Limitations

Although the study is comprehensive, given the sample size, it has some weaknesses. This study was a single-unit study; therefore, the result is not necessarily transferable to other settings. The study did not adjust for changes in patient volume to the SUD emergency ward over time and did not include an analysis of the time for the last visit and time of death. The study did not include a multivariable analysis since it would have hidden the change over time, and a bivariate analysis was chosen as the result showed a non-linear relationship. Statistics could not be retrieved from all patient visits to the emergency ward due to patients without a personal identity number. This group may constitute a risk group but had to be excluded from the study related to limitations within our electronic health records. Even though clinical doctors set the diagnosis following a standardized protocol, the study did not have any validation of the diagnosis set in the SUD emergency ward. Additionally, the study did not include data on the cause of death. Further, in the interpretation of data, we had no information if patients visited the SUD emergency ward voluntarily, through social services or other healthcare providers, police, or by ambulance.

### Future research

Future research on death rate and healthcare visits should focus on the cause of death in relation to demographics, including SUD diagnosis. The time of death after the last healthcare visit could also expand the understanding of risk within patient safety. The study highlights the need for future research on multiple visits for patients with alcohol diagnosis and why increased visits to the emergency ward decrease the death rate for this patient group.

## Conclusion

This study demonstrates that data from visits to SUD emergency wards can be used to identify mortality risk factors, such as gender, type of diagnosis, number of diagnoses, and number of visits to the SUD emergency ward. Knowledge about patterns of patient visits and mortality risk could be used to increase patient safety through a decision support tool integrated with the electronic medical records. An improved system for early detection of increased mortality risk offers an opportunity for an adaptive patient safety system.

## Data Availability

The data supporting this study’s findings are available from Health Care Services Stockholm County. Still, restrictions apply to the availability of these data, which were used under license for the current study and are not publicly available. Data are, however, available from the corresponding author upon reasonable request and with permission of Health Care Services Stockholm County.

## Abbreviations

SUD: Substance Use Disorder
ICD-10: International Classification of Diseases
OAT: Opioid Agonist Treatment
